# Risk factors for cephalic necrosis after plate and screw osteosynthesis of 3- and 4-part proximal humerus fractures: prospective cohort study of 121 patients

**DOI:** 10.1007/s12306-025-00920-x

**Published:** 2025-08-31

**Authors:** Gianluca Canton, Noemi Zaffaroni, Dario Ghassempour, Andrea Marchetti, Antongiulio Favero, Alex Buoite Stella, Gioia Giraldi, Chiara Ratti, Belinda Trobec, Luigi Murena

**Affiliations:** 1 Department of Medicine, Surgery and Health Sciences, University of Trieste, Trieste, Italy; 2 Orthopaedics and Traumatology Unit, Cattinara Hospital – ASUGI, Trieste, Italy

**Keywords:** Proximal humerus fracture, Avascular necrosis, Complications, Osteosynthesis, Risk factors

## Abstract

**Purpose:**

Avascular necrosis (AVN) of the humeral head is a relatively frequent complication after proximal humerus fractures (PHF), often leading to poor outcomes and reoperation. This study investigates both non-modifiable (fracture type, trauma energy, age, sex, Charlson comorbidity index) and modifiable (surgical access, bone graft use, reduction quality) risk factors for post-operative AVN in Neer 3–4-part PHFs.

**Methods:**

Patients with Neer 3–4-part PHFs treated using angular stable plates and followed for at least 6 months were included. Clinical and radiographic elements were evaluated. Statistical analysis was performed with SPSS 26, evaluating the data by both descriptive and univariate analyses.

**Results:**

Among 121 patients (mean age 63, mean follow-up 10.1 months), 8 developed AVN (6.6% incidence). Only 50% of AVN cases occurred within the first 15 months of follow-up. AVN was significantly associated with 4-part fractures (*p* = 0.050), medial hinge disruption (*p* = 0.022), tuberosity comminution (*p* = 0.003), failure to restore the cervico-diaphyseal angle (*p* = 0.022), and residual varus/valgus deformity (*p* = 0.01). The presence of a bone graft suggested a fourfold-increased risk of AVN (OR = 4.219).

**Conclusions:**

The present study confirms that the risk of necrosis is predicted by the type of fracture, as well as by the quality of fixation. Age, sex and CCI of the patient, varus/valgus fracture displacement, glenohumeral dislocation and energy of the trauma did not suggest the risk of AVN. These findings underscore the importance of meticulous surgical techniques to address these factors and potentially reduce the incidence of AVN.

**Level of evidence III:**

Prospective cohort study.

## Introduction

Proximal humerus fractures (PHFs) have a prevalence between 4 and 10% in the general population. They represent the third most frequent fracture in patients over 65, after proximal femur and distal radius fractures. PHFs in the female population are commonly the result of low-energy trauma, while in the male gender there’s a bimodal distribution, being mainly secondary to high-energy trauma between 18 and 60 years old and secondary to low-energy trauma after 65 years old [1, 2]. A key aspect to assess in these injuries is the risk of vascular compromise, which can lead to avascular necrosis (AVN) of the humeral head. This aspect is extremely relevant as it represents in most cases the key factor in the treatment choice between internal fixation and shoulder replacement.

Humeral head vascularisation is guaranteed by branches of the axillary artery, the anterior (35%, ACHA) and the posterior (65%, PHCA) circumflex humeral arteries [3, 4]. In terms of the vascularisation of the joint capsule, the anterior capsular branches are associated with antero-medial fragments, while the posterior capsular branches are associated with postero-medial fragments [5].

Damage to the ACHA occurs in up to 80% of complex PHFs, while the PHCA is intact in 85% of cases. Protection of vascular structures is fundamental during surgery [4]. Concerning Neer 3- and 4-part PHF, there is a wide variability in the reported incidence of AVN of the humeral head, ranging from 0 to 34% of cases [4, 6, 7].

Most studies have been carried out in order to identify the possible predictors of the risk of avascular necrosis. One of the most relevant was published by Hertel et al. in 2004 [8], who found a correlation between distinct characteristics of fracture and AVN. Anatomical neck fracture of the humerus, medial metaphyseal extension of the humeral head less than 8 mm and medial hinge disruption, when combined, resulted in a positive predictive value for AVN of 0.97. The authors also hypothesised that if the anteromedial segment has an extension of more than 8 mm and a joint segment displacement of less than 5 mm relative to the shaft, the perfusion of the joint segment has a limited risk of being compromised [8].

The study by Campochiaro et al. (2015) [9] re-evaluated the parameters found by Hertel. The authors found no correlation with gender, age and type of fracture in a population of patients that presented an AVN incidence of 3.7%. Hertel’s criteria were present in only 30% of the AVN patients, whereas they were present in 4.7% of the non-AVN group. Furthermore, fracture reduction was poor in 50% of the cases in the AVN group, posing emphasis on the importance of the calcar area fragmentation and reduction. The author concluded that Hertel’s criteria are important but not sufficient to predict the necrosis [9]. Time to surgery was also evaluated as a possible risk factor, with no correlation with ANV found in the studies by Boesmueller et al. (2016) and Archer and Furey (2016) [10, 11].

At present, the risk factors for AVN after PHF have not been completely established, and no classification system can certainly be used as a guide for choosing the right surgical treatment in such cases [12–15]. The aim of the present study was to evaluate the correlation between several possible risk factors for post-operative AVN in patients treated with locking plate fixation for 3- and 4-part Neer's PHF.

## Materials and methods

The study population included all adult patients who underwent open reduction internal fixation (ORIF) with angular stable plates for complex (Neer 3- and 4-part) PHF at the Orthopedics and Traumatology Department of the Cattinara Hospital (Trieste, Italy) between December 2014 and December 2021. Inclusion criteria were: age > 18 years at the time of trauma, minimum 6 months of radiographic and clinical follow-up, polytraumatised and non-polytraumatised patients.

A radiographic analysis of the pre-treatment fracture characteristics was performed, both on the X-rays and CT scan, evaluating the number of the fragments, the medial hinge, varus or valgus deformity of the humeral head, the comminution of the tuberosities and glenohumeral dislocation. The time between trauma and surgery and the type of surgical access were registered. The quality of the reduction was assessed on post-op X-rays, evaluating cervical–diaphyseal angle and restoration of the medial hinge if interrupted pre-treatment.

Parameters included in the study are shown in Table 1.

**Table 1:** Parameters and data collected in the study

		Data
Patients’ demographic	Age	Years
	Sex	Male/Female
	Patient comorbidities	Charlson comorbidity index
Fracture characteristic	Fracture type	Radiographic classification (3- or 4-part according to Neer)
	Energy of the trauma	Low energy, High energy
	Interruption of the medial hinge	Missing, present, complete disruption
	Comminution of the tuberosities	Greater, lesser tuberosity, both
	Glenohumeral dislocation	Not present, Anterior, Posterior
Post-op data collected	Surgical approach	Delto-pectoral, Transdeltoid
	Bone graft	Present, Not present
	Restoration of the cervico-diaphyseal angle	Physiological cervico-diaphyseal angle (120–150°), Residual varus/valgus > 20°
	Restoration of the medial hinge	No interruption, Restoration, No restoration
	Time between trauma and surgery	

### Statistics and data analyses

All analyses were performed with SPSS version 26.0. Comparisons between individuals who developed the AVN and those who not developed AVN were performed using the Fisher's exact for the categorical variables and Mann–Whitney *U*-test for the continuous variables. Factors associated with a risk of AVN were identified with a binary logistic regression as well as the influence of the orthopaedic shoulder surgeon background on the surgical outcomes versus the trauma surgeon. Data were reported as odds ratio (OR) at the 95% CI. Statistical significance was defined as *p* < 0.05.

## Results

The final study population included 121 patients of which 79 women (65.3%) and 42 men (34.7%), with a mean age of 63 years (range 30–84; SD: 10.7; median 64) and a mean follow-up time of 10.1 months (range 6–55 months). In the present study, we observed 8 cases of AVN with an incidence of 6.6%. In the first 15 months of follow-up, we observed 50% of all cases of AVN. In detail, AVN occurred after 7 months in two cases while after 12, 15, 29, 31, 32 and 55 months, respectively, in the remaining 6 cases. The complete results are presented in Table 2.

**Table 2:** Patients’ demographics, radiographic and surgical characteristics in AVN and NO-AVN patients. Statistical significant differences between the two groups are shown in bold.

Outcome	NO-AVN	AVN	Sig
	*n* = 113	*n* = 8	
Age [years (IQR)]	64 (56–72)	64 (53–74)	0.774
Time elapsed between trauma and surgery [days (IQR)]	6 (3–9)	5 (3–8)	0.350
Fracture pattern			0.023
3 parts	71	1	
4 parts	42	7	
Sex			0.347
Female	75	4	
Male	38	4	
Charlson comorbidity index (CCI)			0.414
0	9	2	
1	35	0	
2	26	3	
3	24	2	
4	12	1	
5	6	0	
6	1	0	
Interruption of the medial hinge			**0.022**
Missing	56	0	
Present	47	7	
Complete disruption	10	1	
Varus–valgus deformity			0.426
No varus–valgus	6	0	
Valgus	85	5	
Varus	22	3	
Comminution of the tuberosities			**0.022**
No comminution	23	1	
Present	90	7	
Greater tuberosity	58	0	
Lesser tuberosity	3	0	
Both	29	7	
Glenohumeral dislocation			0.831
Not present	108	8	
Anterior dislocation	4	0	
Energy of the trauma			0.965
High	28	2	
Low	85	6	
		0	
Surgical approach			0.761
Deltoid-pectoral approach	79	6	
Transdeltoid approach	34	2	
Bone graft			**0.043**
Not present	81	3	
Present	32	5	
Restoration of cervico-diaphyseal angle 120–150°			**0.022**
No restoration	15	4	
No varus–valgus deformity	2	0	
Restoration	96	4	
Medial hinge restoration			**0.030**
No restoration	11	2	
No interruption of the medial hinge	53	0	
Restoration	49	6	

**Fig. 1:**
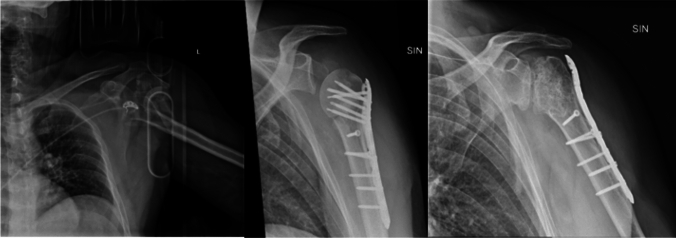
X-rays of a comminuted displaced 4-part proximal humerus fracture in a 47-year-old man following a motor bike accident that led to AVN at 1-year post-op and was treated with proximal screws removal

Correlation with age, CCI, varus or valgus displacement, glenohumeral dislocation, surgical approach, energy of trauma and time between trauma and surgery did not show a statistically significant correlation with AVN (Table 2). Focusing on the fracture type, 3-part fractures accounted for 59.5%, 4-part fractures for 40.5%. There was a statistically significant association between 4-part fractures and AVN (OR = 11.83; 95%CI = 1.407–99.550) (*p* = 0.023) (Fig. 1).

A statistically significant difference emerged when comparing medial hinge integrity versus AVN risk: 87.5% of AVNs occurred in cases of interrupted medial hinge and 12.5% in case of complete hinge disruption (*p* = 0.022). Grouping the data (hinge interruption and disruption), the comparison is even more significant (*p* = 0.007) as all AVNs are in the group of fractures with interrupted/disrupted medial hinge after trauma. The simultaneous comminution of greater and lesser tuberosities was found in 87.5% of AVNs (*p* = 0.022).

**Fig. 2:**
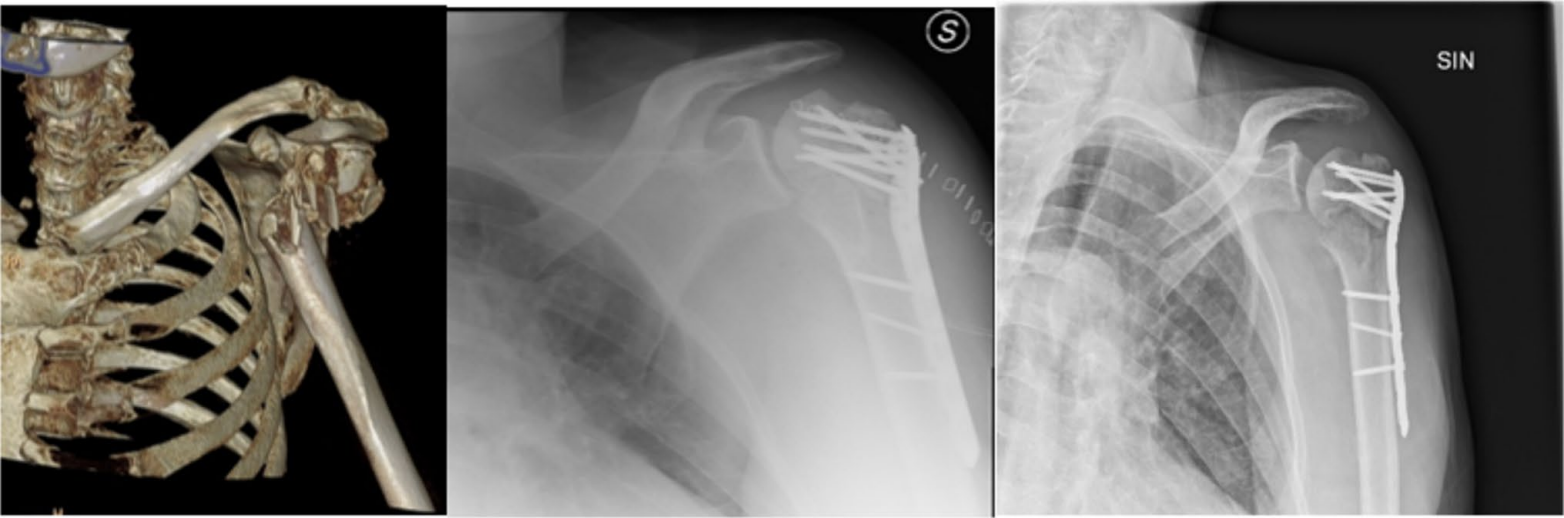
3D-TC and X-rays of a comminuted proximal humerus fracture in a 75-year-old man, pedestrian hit by a car. Inadequate restoration of both the medial hinge and the cervico-dyaphyseal angle, finally leading to AVN after 8 months post-op

Concerning the data collected after surgery, inadequate medial hinge restoration resulted to be significantly correlated to AVN (*p* = 0.030). Likely, inadequate restoration of the cervico-diaphyseal angle showed a statistically significant correlation with AVN (*p* = 0.022) (OR = 6.400, 95%CI = 1.444–28.366). A graft was used in 30.6% of cases, while the percentage raised to 62.5% in the AVN group (*p* = 0.043). The presence of a graft was related to a fourfold increased risk for avascular necrosis (OR = 4.219, 95%C.I. 0.952–18.695) (Fig. 2).

## Discussion

The overall incidence of AVN in the present study was 6.6%, which compares favourably with other literature reports. Indeed, the incidence ranges between 1 and 35% in the literature, but most of the reported results are between 4 and 6% for all PHFs [4, 6, 7]. However, when only 3- and 4-part fractures are concerned, the AVN incidence is slightly higher, with most reports ranging from 11 to 16% [16].

The median time to AVN occurrence, according to the present study, is about 7 months. A similar study published by Spross et al. (2012) reports a median time of 207 days [17]. The correspondence of the two data is intriguing; however it must be emphasised that our data report wide variability in the occurrence of necrosis (four cases within 15 months, the others between 29 and 55 months). These data are a reminder of the intrinsic limits of AVN studies. In fact, AVN can develop a long time after surgery (up to 5 years reported in the literature), and in some cases, symptoms can be very mild, leading to potential underestimation of the actual epidemiology. Indeed, the literature agrees that AVNs are underestimated complications. This consideration applies also to the present study, since minimum X-ray follow-up was 6 months, and only a few patients reached more than 24 months of radiographic follow-up.

Few studies in the literature have looked for a correlation between Charlson comorbidity index and PHF treatment outcomes. Fernández-Cortiñas et al. (2021) report a higher risk of complications and poorer outcomes for patients with a CCI greater than 5, suggesting how general health issues may influence the head vascularity [18]. In this study, this trend is not confirmed; however, the median CCI score in the present study was 2, which is probably too low to affect the AVN risk.

Medial hinge integrity is one of the most investigated factors correlated with the risk of developing AVN since the paper by Hertel et al. was published in 2004 [8]. Many later studies confirmed that medial hinge disruption is a risk factor for humeral head AVN [9, 11, 12]. The present study data definitively confirm this statement, as 100% of AVN cases had medial hinge disruption, with a statistically strong correlation (*p* = 0.007). In predictive terms for AVN, interruption of the medial hinge at the time of injury has stronger statistical evidence than its successful restoration during surgery (*p* = 0.030). Another very interesting finding emerges from the analysis of the cervico-diaphyseal angle restoration after surgery, as inadequate restoration significantly increases the risk of AVN (OR = 6.4). To the best of our knowledge, no other similar conclusions emerge from the literature. Several authors agree on the importance of reconstituting this angle to avoid post-operative complications and worse functional outcomes, but there is no suggested correlation with the risk of cephalic necrosis. It could be hypothesised that poor cervico-diaphyseal angulation prevents the recovery of proper humeral vascularity. Alternatively, it might be considered as a secondary sign of severe instability or technically demanding reduction, potentially leading to further vascular damage to the head. The present study assumes the optimal cervico-diaphyseal angle to be between 120° and 150°. Considering the efforts made in the literature to further subdivide this range and the low significance achieved [19], further subdividing this range would probably not lead to an increase in evidence.

In the present study, 7 out of 8 AVN cases presented with both tuberosities comminution. The possible reason resides in the anatomy of the axillary branches that penetrate the tuberosities to provide nourishment for the head, either through their main trunks or through their anastomotic branches [3, 4]. Nevertheless, several authors correlated the risk of cephalic necrosis with the extent of tuberosity displacement, using a cut-off of 10 mm, whereas tuberosity comminution is less studied [5, 9]. Finally, the use of bone graft significantly increases the risk of AVN in the present study (OR = 4).

The possible explanation might be found in the correlation between bone loss and necrosis. In fact, the use of bone graft in PHF is usually due to severe epiphyseal bone loss, which requires mechanical support to maintain stability [20, 21]. Since compromised bone vascularity plays a key role in AVN development, this could suggest a potential correlation between bone loss and AVN risk, though further research is needed to confirm this association. Moreover, bone graft is more commonly used in more complex fracture types that present other significant risk factors for AVN, such as comminution and hinge disruption. The present study data do not allow to perform a multivariate analysis that would help clarify this aspect.

## Conclusions

The present study confirms a correlation between the risk of necrosis and the type of fracture (4-part fractures, interruption of the medial hinge, comminution of both tuberosities), as well as a correlation with the quality of reduction (medial hinge and varus/valgus alignment restoration) and the use of bone graft. Patient characteristics such as age, sex, and CCI, as well as factors such as varus/valgus fracture displacement, the presence of glenohumeral dislocation, and trauma energy did not correlate with the risk of AVN. Regarding the surgical aspect, the present study does not confirm a correlation between AVN and the time elapsed between trauma and surgery, nor with the type of surgical approach.

## Data Availability

Raw data are available upon reasonable request to the corresponding author.
